# Enhanced Performance of an Au/MoS_2_/GaAs Photodetector by Room-Temperature Metal Electrode Transfer

**DOI:** 10.3390/nano16100624

**Published:** 2026-05-19

**Authors:** Chunxia Li, Weichao Jiang, Cong Qiu, Jingping Xu

**Affiliations:** 1 School of Microelectronic, Shenzhen University of Information Technology, Shenzhen 518172, China; licx@suit-sz.edu.cn (C.L.); qiucong@suit-sz.edu.cn (C.Q.); 2 School of Integrated Circuits, Huazhong University of Science and Technology, Wuhan 430074, China

**Keywords:** MoS_2_, GaAs, photodetector, transferring electrode

## Abstract

Recently, self-powered MoS_2_/GaAs photodetectors have attracted intensive attention. However, thermal processing following metal–electrode deposition tends to damage the lattice structure of MoS_2_, leading to degraded device performance and poor consistency. In this work, Au/MoS_2_/GaAs photodetectors are fabricated using two different methods of transferring Au (Tr-Au) and thermal evaporation Au (TE-Au), and their photoelectric performances are compared. It is found that, compared to TE-Au devices, the Tr-Au devices exhibit higher responsivity (45.29 A/W) and detectivity (3.11 × 10^13^ Jones). The underlying mechanisms are attributed to a significant reduction in defect traps in MoS_2_ and a smooth MoS_2_/GaAs heterojunction interface, which collectively increase photocurrent and suppress dark current. Therefore, the room-temperature Au transfer method shows great promise for the fabrication of high-performance optoelectronic devices.

## 1. Introduction

Since the discovery of two-dimensional (2D) materials such as graphene in 2004 [1], extensive research has been conducted in this field [2,3,4,5]. Compared to bulk materials, 2D materials possess a unique lattice structure that makes them more sensitive to electric fields, optical fields and strain effects, therefore they hold great promise for optoelectronic applications [6,7,8,9,10]. Among them, MoS_2_, a representative transition metal dichalcogenide, stands out due to its high photoelectric gain, excellent carrier mobility and tunable bandgap (1.2~1.8 eV) [6,7,8]. Moreover, large-scale CVD growth of MoS_2_ has already been achieved, positioning it as an ideal candidate for visible to near-infrared photodetection. However, the weak light absorption (~5% for monolayer MoS_2_) [5] and high dark current [9], which lead to low detectivity, have hindered the development of MoS_2_-based photodetectors. Considerable efforts have been devoted to improving the quality of MoS_2_-based photodetectors through various approaches, including MoS_2_/bulk-semiconductor heterojunctions [10,11], MoS_2_-based transistors [12,13], and novel 2D materials [14,15]. Among these, MoS_2_/GaAs heterojunctions have attracted particular attention due the superior properties of GaAs, such as its high electron mobility, suitable effective mass and wider bandgap, which makes it a highly favored second-generation semiconductor. Li et al. demonstrated that MoS_2_/GaAs heterojunctions an enhance light absorption and utilize the built-in electric fields to efficiently separate photogenerated electron–hole pairs, enabling self-powered, and high-performance photodetectors [10]. Lin et al. introduced hexagonal boron nitride (h-BN) into MoS_2_/GaAs heterojunctions to improve power conversion efficiency [11]. However, previous studies have often relied on high-work-function metals such as Au or Pt as contact electrodes, which require deposition processes involving high-temperature thermal evaporation or high-energy electron beam bombardment [15]. These methods typically damage the lattice structure of MoS_2_, resulting in degraded device performance and poor consistency.

In this work, to address this issue, a low-temperature metal–electrode transfer technique is adopted to replace conventional high-energy or high-temperature fabrication methods for preparing Au/MoS_2_/GaAs photodetectors. By preserving the lattice integrity of MoS_2_ and ensuring a smooth MoS_2_/GaAs interface, the resulting device achieves a responsivity of 45.29 A/W and a detectivity of 3.11 × 10^13^ Jones, nearly a 250% improvement over those with thermally evaporated electrodes. Statistical analyses further confirm excellent device uniformity. This work offers a straightforward and effective route toward high-performance 2D optoelectronic devices.

## 2. Materials and Methods

**Figure 1:**
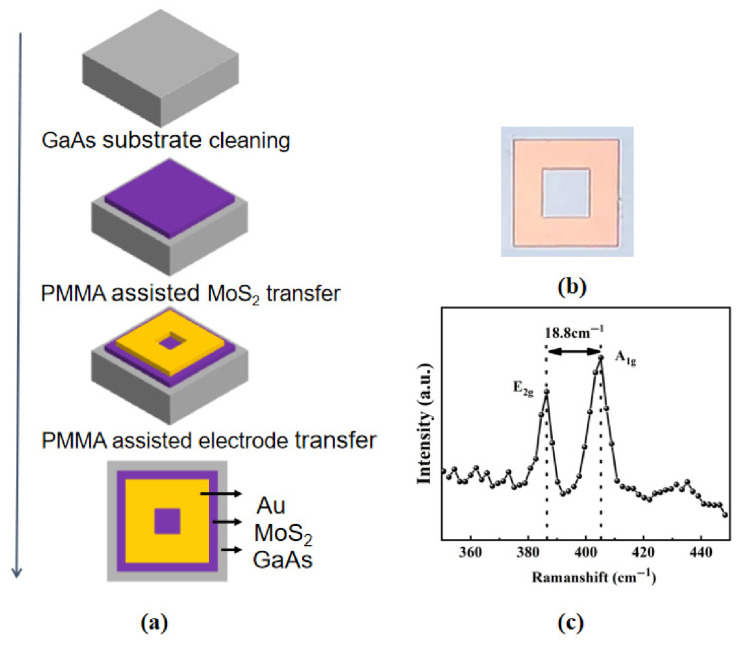
(**a**) Fabrication process flow of the Au/MoS_2_/GaAs photodetector; (**b**) optical microscopy image of the fabricated devices; (**c**) Raman spectrum of the MoS_2_ layer on the GaAs substrate.

Figure 1a illustrates the fabrication process flow of the Au/MoS_2_/GaAs photodetector using low-temperature Au-electrode transfer. An n-type GaAs wafer with a doping concentration of 10^18^ cm^−3^ was used as the substrate. The wafer was first rinsed with deionized (DI) water, followed by sequential ultrasonic cleaning in acetone, ethanol and isopropanol for 5 min each to remove organic residues and surface impurity particles. It is important to note that the ultrasonic power should be kept as low as possible to avoid wafer fragmentation. The wafer was then dipped in a diluted HF solution (4~5%) for 5 min to remove the native oxides. Subsequently, it was soaked in an 8% ammonium sulfide (NH_4_)_2_S solution for 40 min at room temperature to eliminate surface oxides and prevent re-oxidation through sulfur passivation. After that, the GaAs wafer was rinsed with DI water 4~5 times to remove the residual ammonium sulfide solution and dried with N_2_ gas. Next, a monolayer CVD (chemical vapor deposition)-grown MoS_2_ thin film (purchased from Six Carbon Corporation, Shenzhen, China) was transferred onto the GaAs surface with the assistance of a PMMA supporting layer [16]. A similar method was used to transfer a pre-deposited Au electrode film (~40 nm thick, 30 × 30 µm^2^ in area) onto the MoS_2_ surface, and the resulting sample is denoted as the Tr-sample. The Au-electrode transfer process is detailed as follows: (1) first, Au was evaporated onto a SiO_2_/Si substrate and patterned into electrodes using a lift-off process; (2) the substrate was then spin-coated with PMMA at 3000 rpm for 60 s, followed by baking on a hot plate at 150 °C; (3) subsequently, the wafer was dipped in a 4.12 mol/L HF solution to etch away the SiO_2_ layer, causing the PMMA-supported Au electrode to detach from the Si substrate; (4) the PMMA/Au electrode film was then transferred to the DI water to remove the residual HF; (5) the MoS_2_/GaAs wafer was aligned with the PMMA/Au film by naked eye in DI water to form the PMMA/Au/MoS_2_/GaAs structure, then the assembly was dried with N_2_ gas, followed by sequential baking at 80 °C for 600 s and at 150 °C for another 600 s; and (6) finally, the PMMA sacrificing layer was removed by immersion in acetone for 20 min at room temperature. For comparison, a control sample (denoted as the TE-sample) was fabricated by directly thermally evaporating Au onto the MoS_2_ surface at room temperature under a high vacuum of ~10^−5^ Torr, with an evaporation rate of 0.01~0.03 nm/s. The Au film was then patterned into electrodes with an area of 30 × 30 µm^2^ via a lift-off process. Both the Tr- and TE-samples exhibit identical optical micrographs, as shown in Figure 1b.

Figure 1c presents the Raman spectrum of the MoS_2_ flake on the GaAs substrate. Two characteristic Raman peaks, corresponding to the A_1g_ and E_2g_ modes of MoS_2_, are observed at 405.2 cm^−1^ and 386.4 cm^−1^, respectively. The Raman shift between the A_1g_ and E_2g_ is 18.8 cm^−1^, confirming that the transferred MoS_2_ flake is monolayer, which is consistent with previous reports [8,17].

## 3. Results and Discussion

### 3.1. Operating Principle of the Au/MoS_2_/GaAs Photodetector

**Figure 2:**
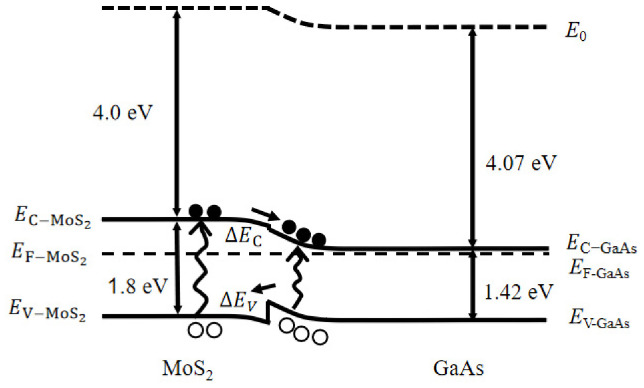
Energy band diagram of the MoS_2_/n-GaAs heterojunction under light illumination.

Figure 2 shows the energy-band diagram of the MoS_2_/n-GaAs heterojunction under light illumination. The electron affinities and bandgap of GaAs and MoS_2_ are 4.07 eV/1.42 eV and 4.0 eV/1.80 eV, respectively [18,19,20]. The Fermi level of MoS_2_ ($E_{F\text{-}MoS_{2}}$) lies slightly above the mid-gap, as monolayer MoS_2_ is typically n-type without intentional doping [21]. In contrast, the Fermi level of GaAs (E_F-GaAs_) is close to its conduction band (E_C-GaAs_) due to heavy doping. When the MoS_2_ flake is transferred onto the n^+^-GaAs substrate, the majority carriers (electrons) diffuse from n^+^-GaAs into MoS_2_, establishing a built-in electric field across the MoS_2_/GaAs interface. This results in upward band bending in GaAs and downward band bending in MoS_2_, as illustrated in Figure 2. The conduction-band offset (ΔE_C_) is 0.07 eV, while the valance-band offset (ΔE_V_) is 0.31 eV. Under equilibrium conditions, the barrier height is roughly estimated to be ~0.83 eV, based on the electron affinity difference (0.07 eV) and the Fermi level difference (~0.9 eV) between n^+^-GaAs and monolayer MoS_2_. Upon light illumination, both MoS_2_ and GaAs absorb photons to generate electron–hole pairs. Driven by the built-in field, the photogenerated electrons and holes flow from MoS_2_ to GaAs and from GaAs to MoS_2_, respectively, thereby producing a photocurrent.

### 3.2. Photoelectric Performance of TE-Sample

**Figure 3:**
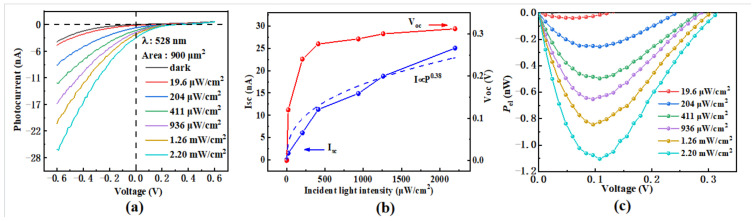
(**a**) *I*-*V* characteristics of the control sample in the dark and under 528 nm laser illumination at various light intensities; (**b**) extracted open-circuit voltage (*V*_oc_) and short-circuit current (*I*_sc_) as functions of light intensity at zero bias, where the blue dashed line represents the power-law fit; and (**c**) electrical power as a function of bias voltage.

First, the photoelectric performance of the control sample (TE-sample) was investigated. To evaluate the influence of light intensity on device performance, the photocurrent–voltage (*I*-*V*) curves of the control sample were measured under 528 nm laser irradiation at various light intensities ranging from 0 (dark condition) to 2.2 mWcm^−2^, as shown in Figure 3a. The asymmetric *I*-*V* curves indicate a rectifying behavior characteristic of the MoS_2_/GaAs heterojunction photodetector, which exhibits a clear response to 528 nm illumination [17,22,23]. As clearly demonstrated in Figure 3a, the photocurrent increases with rising light intensity, which can be attributed to the increased number of photogenerated carriers under stronger illumination [24]. To further investigate the effect of the light intensity on the illumination characteristics of the TE-sample, the short-circuit current (*I*_sc_), open-circuit voltage (*V*_oc_), and the electrical power (*P*_el_) were extracted and are plotted in Figure 3b and Figure 3c, respectively. Figure 3b shows that a notable *I*_sc_ exists even at zero bias, which is attributed to the drift of photogenerated electrons and holes across the barrier region driven by the built-in field [22]. Moreover, *I*_sc_ increases as a power law with the light intensity, due to the increased density of photogenerated carriers crossing the barrier region. The relationship between the photocurrent and incident light intensity can be expressed as $I_{ph}={cP}^{\alpha }$, where *I*_ph_ is the photocurrent, *P* is the incident light intensity, *c* is a proportionality constant dependent on the wavelength, and α is the power-law fitting exponent [24,25]. As mentioned above, *I*_sc_ corresponds to the photocurrent *I*_ph_ at zero bias under 528 nm illumination. By fitting the *I*_sc_ curves using the power-law function, the exponent α is determined to be approximately 0.38 (<1) for the TE-devices. This value, being significantly less than unity, indicates the presence of structural defects in MoS_2_ or interface states at the MoS_2_/GaAs heterojunction, which reduce the collection efficiency of photogenerated carriers during illumination [25]. In contrast, an α value equal to or very close to 1 would signify perfect collection of photogenerated carriers and an ideal, defect-free MoS_2_/GaAs heterojunction [22]. When the devices are illuminated, these defects or interface states tend to trap photogenerated carriers, thereby affecting the local electrostatic field at the MoS_2_/GaAs heterojunction and consequently influencing the *V*_oc_. This phenomenon is known as the photogating effect [26]. As shown in Figure 3b, the *V*_oc_ of the TE device shifts positively and increases from 0 to 0.312 V as the light intensity increases from 0 to 2.2 mW/cm^2^. This increase occurs because a higher incident optical intensity leads to more photogenerated carriers being separated and transported by the built-in field, resulting in an elevated *V*_oc_. Nevertheless, a saturation tendency in *V*_oc_ is observed in Figure 3b. A plausible explanation that the structural defects in MoS_2_ or the interface states at the heterojunction become gradually filled as the light intensity increases, which modulates the built-in field and causes *V*_oc_ to approach a saturation value [24,27]. As shown in Figure 3c, the electrical power (*P*_el_) is strongly dependent on both the applied voltage and the light intensity, suggesting that the optimal output performance of the device can be achieved by selecting appropriate values for these parameters. For example, an input voltage of 0.1 V and a light intensity of 2.2 mW/cm^2^ yield an electrical power of approximately 1.1 nW.

**Figure 4:**
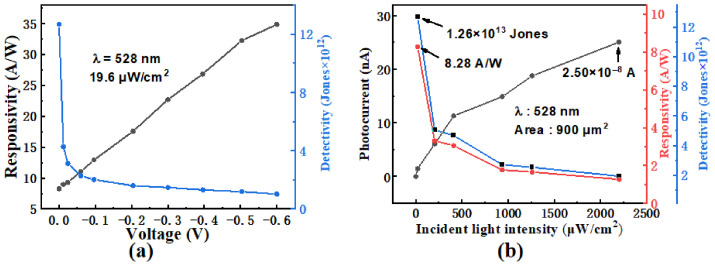
(**a**) Responsivity and detectivity of the control samples measured under various bias voltages; (**b**) photocurrent, responsivity and detectivity of the control samples as functions of incident light intensity at zero bias voltage.

Responsivity (*R*) and detectivity (*D*) are two crucial parameters for evaluating photodetectors, defined by the following equations [28,29,30]:

(1) $$R=\frac{I_{ph}-I_{D}}{PA}$$

(2) $$D=\frac{A^{1/2}\times R}{{\left(2qI_{D}\right)}^{1/2}}$$

where *I*_D_, *P*, *A,* and *q* represent the dark current, incident light intensity, device area, and electron charge, respectively. Figure 4a represents the responsibility and detectivity of the control device under 528 nm laser illumination at various bias voltages. The responsivity increases with bias voltage, which is attributed to the corresponding increase in photocurrent. In contrast, the detectivity decreases sharply with bias voltage, as the dark current becomes large under higher bias voltage, as evident in Figure 4a. Figure 4b shows the photocurrent, responsivity, and detectivity of the control samples under different incident light intensities at zero bias. Under 528 nm laser illumination with a light intensity of 19.6 μW/cm^2^ at zero bias, the TE device exhibits self-powered behavior, achieving a maximum responsivity of 8.28 A/W and a maximum detectivity of 1.26 × 10^13^ Jones. This indicates an efficient generation and collection of electron–hole pairs under weak illumination. As the light intensity increases, both responsivity and detectivity decrease sharply and then gradually approach saturation. This behavior can be explained by the photogating effect: under high light intensity, the electric field in the space charge region is reduced due to carrier trapping at band tail states of MoS_2_ or at the MoS_2_/GaAs interface. These trap states become progressively saturated by photogenerated carriers [7,17], thereby limiting the collection of photogenerated carriers and reducing detectivity [26]. In addition, the photocurrent is observed to increase with increasing light intensity, which is attributed to the higher density of photogenerated carriers resulting from stronger illumination.

### 3.3. Photoelectric Performance of Tr-Sample

**Figure 5:**
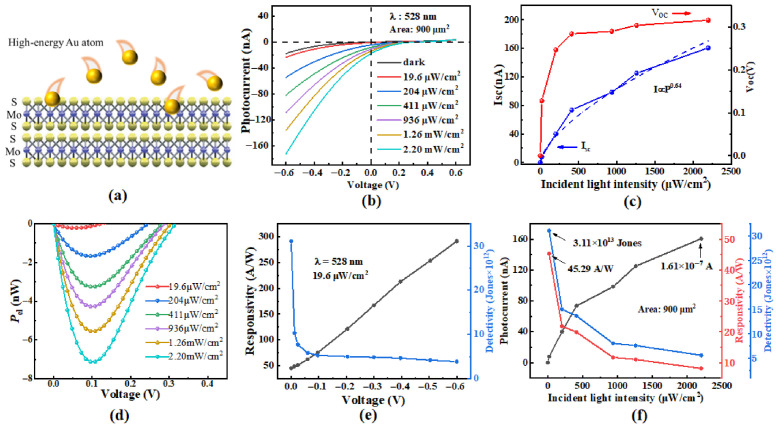
(**a**) Schematic diagram illustrating lattice damage in MoS_2_ induced during thermal evaporation; (**b**) *I*-*V* curves of the transferred-Au/MoS_2_/n-GaAs photodetector in the dark and under 528 nm laser irradiation; (**c**) the extracted open-circuit voltage and short-circuit current as functions of light intensities, where the blue dash line represents the power-law fit; (**d**) electrical power as a function of bias voltage; (**e**) responsivity and detectivity of the devices under various bias voltages; and (**f**) photocurrent, responsivity, and detectivity of the devices under different incident light intensities at zero bias.

For the control sample, the high-energy Au atoms generated during thermal evaporation can damage the MoS_2_ lattice, as schematically illustrated in Figure 5a. This damage introduces traps that capture photogenerated carriers and degrades the quality of the MoS_2_/GaAs heterojunction, thereby deteriorating the device performance. To address this issue, an improved strategy involving the transfer of a pre-deposited Au film onto the MoS_2_ surface was adopted, and the corresponding performance of the transferred device (Tr-device) was characterized. Figure 5b shows is its *I*-*V* curves measured in the dark and under 528 nm laser irradiation. Compared with the results shown in Figure 3a, the photocurrent of the Tr-device is significantly enhanced, indicating improved photovoltaic performance [30]. Because the transfer process induces less lattice damage to MoS_2_, the density of trapping sites for photogenerated carriers is greatly reduced. Consequently, more carriers can be swept across the space-charge region and contribute to the photocurrent, leading to a substantial increase in photocurrent [22,24].

Figure 5c presents the extracted *I*_sc_ and *V*_oc_ of the Tr-devices. Compared with the TE-sample, the Tr-device exhibits a higher *I*_sc_ and an enhanced self-powered photovoltaic performance, which can be attributed to the improved quality of the MoS_2_/GaAs heterojunction, as discussed above. Moreover, the fitted power-law exponent α for the Tr-device is 0.64, which is larger than that of the TE-sample, indicating fewer structural defects at the MoS_2_/GaAs interface and further supporting the improved heterojunction quality. Nevertheless, the α value remains below unity, suggesting that a substantial number of defects still exist within the MoS_2_ layer. These residual defects are likely due to intrinsic structural imperfections, such as sulfur vacancies, which are inevitably introduced during chemical vapor deposition [31]. As shown in Figure 5c, the *V*_oc_ of the Tr-device increases from 0 V to 0.32 V as the light intensity rises from 0 to 2.2 mW/cm^2^, which is slightly higher than that of the TE-sample. Both the built-in voltage and the interfacial charges at the MoS_2_/GaAs interface are believed to contribute to the observed *V*_oc_ [32]. As previously noted, high-temperature Au evaporation can induce lattice damage in MoS_2_, creating defects that may act as the minority carrier traps [24,33]. When the TE-sample is illuminated, minority traps capture photogenerated holes in the negative space-charge region (within MoS_2_), partially neutralizing the negative charges in that region and thereby reducing the build-in voltage and *V*_oc_. In contrast, the Tr-device exhibits a higher *P*_el_ than the TE-sample under various forward bias voltages, as shown in Figure 5d, indicating a stronger photovoltaic effect.

Figure 5e presents the *R* and *D* of the Tr-Au/MoS_2_/GaAs device under 528 nm laser illumination at different bias voltages. Comparing Figure 5e with Figure 3c, both *R* and *D* of the Tr-device are significantly higher than those of the TE-sample, primarily due to con the improved quality of the MoS_2_/GaAs heterojunction. According to Equation (1), the higher R of the Tr-device results from its increased photocurrent. The higher *D* indicates a superior ability to detect weak optical signals compared to the TE-sample, which is attributed to the reduced trap density and the enhanced *R*.

Figure 5f shows the photocurrent, responsivity and detectivity of the Tr-device under various incident light intensities at zero bias. For clarity, the extracted value of photocurrent, responsivity and detectivity for both samples are summarized in Table 1. Compared with the TE-sample, the Tr-device exhibits superior photoelectric performance, with a detectivity of 3.11 × 10^13^ Jones, a responsivity of 45.29 A/W, and a photocurrent of 1.61 × 10^−7^ A.

**Figure 6:**
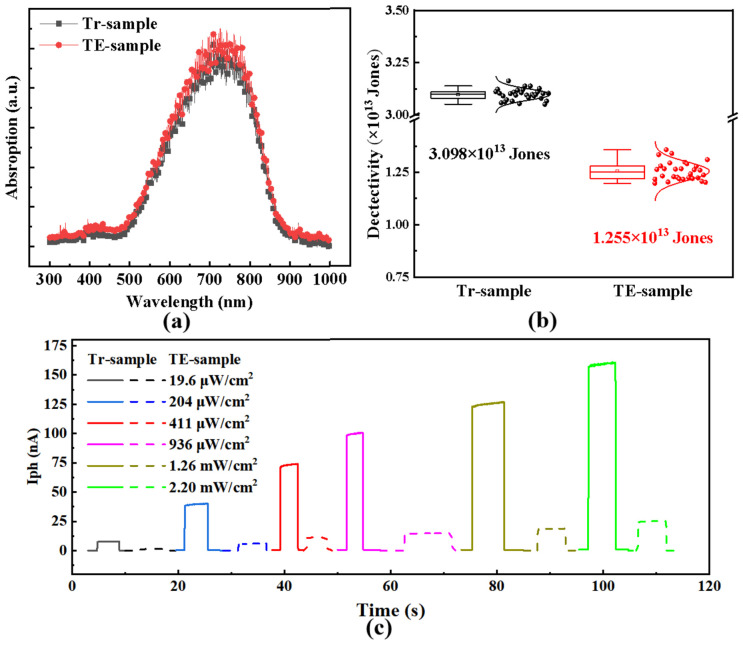
(**a**) Absorption spectra of monolayer MoS_2_ for the thermally evaporated Au (TE) and transferred Au (Tr) samples; (**b**) normal distributions and box plots of the *D* extracted from the photocurrents of 30 devices for the Tr- and TE-samples; (**c**) time-resolved photoresponse of the Tr- and TE-samples under incident light intensities ranging from 19.6 µW/cm^2^ to 2.2 mW/cm^2^ at zero bias voltage, with an on/off period of 10 s.

To investigate the influence of thermally evaporated (TE) and transferred (Tr) Au contacts on the photoelectric performance, the absorption spectra, statistical distribution of detectivity *D*, and time-resolved photocurrent response were further measured and are presented in Figure 6. Figure 6a shows the absorption spectra of the TE- and Tr-samples. Both samples exhibit a broad detection wavelength range from 500 to 850 nm and share the same absorption peak at 750 nm. Notably, the Tr-sample shows a slightly higher absorption rate than the TE-sample, suggesting its potential for wide-spectrum photodetection capability [24]. Although the absorption peak is located at 750 nm, electrical characterizations were carried out at 528 nm. This wavelength corresponds to a stable, well-calibrated light source, enabling reliable and repeatable evaluation of key device parameters such as responsivity, dark current, and switching behavior. Figure 6b presents the normal distributions and box plots of detectivity extracted from the photocurrents of 30 devices. The mean detectivity values are 3.098 × 10^13^ Jones for the Tr-sample and 1.255 × 10^13^ Jones for the TE-sample, indicating improved light detectivity for the Tr-device. The box plots further reveal that the Tr-sample exhibits narrower statistical dispersion and better consistency, suggesting superior and more reliable photoelectric performance.

Figure 6c presents the *I*_ph_ under different incident light intensities over one on/off cycle. As expected, the photocurrent increases with rising light intensity. Compared with the TE-sample, the Tr-sample shows a substantially higher photocurrent, indicating a marked reduction in the trapping of the photogenerated carriers. This improvement is attributed to the absence of lattice damage in MoS_2_, thanks to the elimination of high-energy Au atom bombardment during the transfer process [33]. Notably, the Tr-sample exhibits a pronounced increase in photocurrent upon light illumination, which is likely associated with reduced carrier trapping at the MoS_2_/GaAs heterojunction. This enables a rapid response to light excitation and further confirms the enhanced photoelectric performance of the Tr-device.

## 4. Conclusions

In this work, by transferring a pre-deposited Au film onto the MoS_2_ surface, the integrity of the MoS_2_ lattice is preserved, and a smooth MoS_2_/GaAs interface is achieved. The resulting Tr-Au/MoS_2_/GaAs photodetector exhibits a responsivity of 45.29 A/W and a detectivity of 3.11 × 10^13^ Jones, demonstrating substantially improved photodetection performance compared to devices featuring thermally evaporated Au electrodes. This enhancement contributed to the reduced trap density within the MoS_2_ layer and the increased collection efficiency of photogenerated carriers, both of which are closely associated with the non-destructive integration of two-dimensional materials and metal electrodes. Moreover, statistical analyses confirm the excellent consistency of the photodetection performance, verifying the reproducibility of the proposed Au transfer method. These findings highlight that non-destructive metal integration is critical for precise interface control and defect reduction in the development of high-quality heterojunction devices combining two-dimensional and bulk semiconductors. Collectively, this work not only provides an effective route for fabricating high-performance MoS_2_/GaAs photodetectors but also offers valuable insights for optimizing future fabrication processes of high-performance two-dimensional optoelectronic devices, thereby holding significant potential for the eventual commercial application of 2D/bulk semiconductor heterojunction devices.

## Figures and Tables

**Table 1:** Extracted photoelectric parameters from Figure 5f for Tr- and TE-samples.

Samples	*I*_ph_ (A)	*R* (A/W)	*D* (Jones)
TE-Sample	2.50 × 10^−8^	8.28	1.26 × 10^13^
Tr-Sample	1.61 × 10^−7^	45.29	3.11 × 10^13^

## Data Availability

The original contributions presented in this study are included in the article. Further inquiries can be directed to the corresponding author.
